# A Rasch analysis of the fear of coronavirus-19 scale in South Africa

**DOI:** 10.4102/sajip.v47i0.1861

**Published:** 2021-06-29

**Authors:** Sergio L. Peral, Brandon Morgan, Kleinjan Redelinghuys

**Affiliations:** 1 Department of Industrial Psychology and People Management, College of Business and Economics, University of Johannesburg, Johannesburg, South Africa

**Keywords:** coronavirus-19, fear of coronavirus-19 scale, South Africa, Rasch partial credit model, reliability, validity

## Abstract

**Orientation:**

Investigating the psychological aspects associated with the coronavirus disease might be important for psychological interventions. The fear of coronavirus-19 scale (FCV-19S) has emerged as a popular measure of coronavirus-19-related fear. However, its psychometric properties remain unknown in South Africa.

**Research purpose:**

This study set out to investigate the internal validity of the FCV-19S in the South African context using the Rasch measurement model.

**Motivation for the study:**

There have been some mixed findings on the psychometric properties of the FCV-19S in international research and its psychometric properties are yet to be investigated in South Africa. Investigating these psychometric properties can provide psychometric information to practitioners who wish to use this instrument in the South African context.

**Research approach/design and method:**

A cross-sectional survey research design was used. The FCV-19S was administered to 159 adults. The Rasch partial credit model was applied to the item responses to investigate the measurement quality of the FCV-19S.

**Main findings:**

The FCV-19S showed somewhat satisfactory internal validity in the South African context within the boundaries of the current sample, and clarity was obtained on the mixed findings obtained in the previous research. Potential shortcomings of the scale were identified that might reduce its applicability to the South African context.

**Practical/managerial implications:**

Our results provide tentative support for the internal validity of the FCV-19S in South Africa. Suggestions for the improvement of the scale are made.

**Contribution/value-add:**

This is one of the first studies to investigate the internal validity of the FCV-19S in South Africa. Our results hold important implications for the continued use of this scale and have helped to clarify some of the mixed findings obtained in previous research.

## Introduction

### Orientation

This study set out to investigate the internal validity of the fear of coronavirus-19 scale (FCV-19S) in South Africa using the Rasch (1960) measurement model. Ahorsu et al. (2020) developed the FCV-19S to measure fear associated with the coronavirus-19. They argue that the instrument can be used by healthcare practitioners when developing interventions to help people deal with their fears about the coronavirus-19. Ahorsu et al. (2020) showed that the FCV-19S was a reliable and valid instrument in their sample. Several studies globally have subsequently investigated the psychometric properties of the FCV-19S (e.g. Caycho-Rodríguez, et al. 2020; Martínez-Lorca, Martínez-Lorca, Criado-Álvarez, & Armesilla, 2020; Wakashima et al., 2020), showing mostly satisfactory psychometric properties. However, there have been some mixed results. These mixed results cannot be ignored because they might call into question the reliability and validity of the FCV-19S-scale scores when used in practical settings.

The FCV-19S was developed as a unidimensional instrument. This means that the item responses can be summed to form a single score. Some studies have found evidence for multidimensionality though (Bitan et al., 2020; Reznik, Gritsenko, Konstantinov, Khamenka, & Isralowitz, 2020), suggesting that summated scores might potentially consist of two separate dimensions. This said, the statistical decisions used to find this multidimensionality have some concerns (Pakpour, Griffiths, & Lin, 2020). This makes it difficult to determine if the multidimensionality is real or a statistical artefact. Some studies have applied item response theory (IRT) to the FCV-19S item responses. The IRT has several advantages over and above classical test theory approaches that allow for a more fine-grained analysis of psychometric properties (see Embretson & Reise, 2000, for a discussion of these advantages). However, we noticed that some authors tended to not take full advantage of IRT in their analyses because they tended to focus on overall item locations and overall item model fit. We believe that there is much to be gained by also investigating local dependence of item responses, category functioning of the item responses and test information to obtain a better picture of the reliability and validity of the summated FCV-19S-scale scores.

### Research purpose and objectives

To address these concerns, we applied the Rasch partial credit model (PCM) (Masters, 1982) to investigate the fit of the FCV-19S items to the Rasch measurement model (i.e. internal validity) in South Africa. We included an investigation of (1) the dimensionality of the item scores, (2) local dependence and (3) item and test information to address the concerns about dimensionality and to better understand the measurement quality of the FCV-19S items. The results from our study potentially hold important implications for refinement of the FCV-19S and might shed some insight into the use of the FCV-19S in South Africa. Without this information, practitioners cannot be certain if the FCV-19S-scale scores is usable (i.e. reliable and valid) in the South African context.

## Literature

### The novel coronavirus-19

The novel coronavirus-19 is a disease caused by the severe acute respiratory syndrome coronavirus 2 (SARS-CoV-2) (World Health Organization, 2020). It was declared a global public health emergency by the World Health Organization in January 2020. The Center for Systems Science and Engineering at Johns Hopkins University (2021) indicates that there have been approximately 140 million diagnosed cases of coronavirus-19 and 3 million deaths from the SARS-CoV-2 globally (as reported on the 18th of April 2021). In South Africa, there are approximately 1.5 million diagnosed cases of coronavirus-19 and 53 000 deaths (The Center for Systems Science and Engineering at Johns Hopkins University, 2021). The daily number of new infections globally peaked in July 2020, and a resurgence appeared in December of 2020 (The Center for Systems Science and Engineering at Johns Hopkins University, 2021). In December 2020, the president of South Africa reintroduced stricter lockdown measures to avoid the resurgence of coronavirus-19 in South Africa (Government of South Africa, 2020).

### Psychological impact of coronavirus disease 2019

According to Ahorsu et al. (2020), most of the coronavirus-19 research to date has focused on the medical aspects such as the symptoms of the disease (e.g. Cao et al., 2020; Fauci, Lane, & Redfield, 2020). These symptoms include, for example, lethargy, dry cough, elevated body temperature, sore throat, shortness of breath and gastrointestinal problems (World Health Organization, 2020). This does not imply that no research exists on the psychological consequences of the disease, especially as it pertains to lockdowns and social distancing associated with prevention of the spread of the SARS-CoV-2 (e.g. Chew, Wei, Vasoo, Chua, & Sim, 2020; Talevi et al., 2020). Ammar et al. (2020), for example, found that mental well-being decreased during lockdown periods. There is also evidence that throughout 2020, people experienced more negative emotions and fewer positive emotions because of coronavirus-19 (Arora et al., 2020; Li, Wang, Xue, Zhao, & Zhu, 2020; Serafini et al, 2020). Arora et al. (2020; also see Serafini et al., 2020 for a review) conducted a meta-analysis of observational studies on psychological outcomes of coronavirus-19 and found that fear and worry (i.e. anxiety) were common outcomes associated with the disease.

In South Africa, Kim, Nyengerai and Mendenhall (2020) found that there was a positive relationship between perceived risk of contracting coronavirus-19 and depression after controlling for relevant demographic characteristics. In the qualitative component of their study, they found that most participants did not believe that the coronavirus-19 affected their mental health although some of the participants did report stress, anxiety and fear related to the disease and the lockdowns that were used to prevent spread of the disease. Mbunge (2020) conducted a literature search on the effects of coronavirus-19 in South Africa and argued that the disease and its associated symptoms can lead to stigmatisation and reduced mental health (e.g. anxiety, fear and depression). It is therefore clear that coronavirus-19 has both physical and psychological implications (e.g. Fofana, Latif, Sarfraz, Bashir, & Komal, 2020; Schimmenti, Starcevic, Giardina, Khazaal, & Billieux, 2020).

### Fear of coronavirus-19 scale

The FCV-19S (Ahorsu et al., 2020) is a unidimensional scale consisting of seven items, which use a five-point Likert-type scale response format ranging from *strongly disagree* to *strongly agree*. A summated score is obtained from these responses with a minimum of seven and a maximum of 35. Ahorsu et al. (2020) suggest that this summated score can be used as an indicator of the severity of fear that a person has towards coronavirus-19. This holds true only if the summated score is reliable and valid. The FCV-19S was developed and validated on a sample of 717 participants from Iran. Classical test theory and IRT were used to select the final items included in the scale. Before discussing these psychometric properties, it is important to note that the FCV-19S is not the only scale that can be used to measure psychological attitudes towards the disease (see Cortez, Joseph, Das, Bhandari, & Shoib, 2020). Other scales include, for example, the coronavirus disease 2019 (COVID-19) stress scale (Taylor et al., 2020), the coronavirus anxiety scale (Lee, 2020) and the COVID-19 phobia scale (Arpaci, Karatas, & Baloğlu, 2020), although it appears that the FCV-19S scale has received the most research attention with 864 Google Scholar citations on the 14th of April 2021. Its popularity could possibly be ascribed to the briefness of the measure in comparison to other instruments and its exclusive focus on fear (for those practitioners who are only concerned with the psychological aspect of fear).

### Psychometric properties of the fear of coronavirus-19 scale

The psychometric properties of the FCV-19S have been investigated in Europe (e.g. Iversen et al., 2021; Reznik et al., 2020; Soraci et al., 2020; Tsipropoulou et al., 2020), North America (e.g. García-Reyna et al., 2020; Perz, Lang, & Harrington, 2020), South America (e.g. Andrade et al., 2020; Huarcaya-Victoria, Villarreal-Zegarra, Podestà, & Luna-Cuadros, 2020), Asia (e.g. Chang, Hou, Pakpour, Lin, & Griffiths, 2020; Doshi, Karunakar, Sukhabogi, Prasanna, & Mahajan, 2020), Australasia (Winter et al., 2020) and the Middle East (e.g. Alyami, Henning, Krägeloh, & Alyami, 2020; Haktanir, Seki, & Dilmaç, 2020).^1^ In this section, we briefly review the development of the FCV-19S and then focus on some of the results that have been obtained in other studies. It must be kept in mind that most studies have used translated versions of the FCV-19S, and the results therefore do not speak directly to the English version of the instrument.^2^

Ahorsu et al. (2020) developed the FCV-19S by first examining existing measures of fear and retaining 28 relevant items from these existing measures for the instrument. Two expert review panels were conducted leaving 10 items. A small pilot study was then conducted to determine if participants could understand the items. A sample of 717 adults was subsequently obtained to investigate the psychometric properties of the scale. Three items showed almost zero-corrected item-total correlation coefficients and were therefore removed from the scale. In the Rasch analysis, none of the items showed infit or outfit mean squares > 1.30 or < 0.70 (see the Method section of our article for an explanation of these fit indices). Item I1 (most afraid) did, however, show some misfit with an infit and outfit mean square of 1.26 and 1.25. With respect to item locations, items I1 and I5 (nervous or anxious) were the most difficult items to endorse and items I4 (losing my life) and I7 (heart races) were the easiest to endorse.

### Dimensionality

Although the FCV-19S was designed to be a unidimensional instrument, there have been some conflicting results with respect to its dimensionality. Most studies have found support for one dimension in the item responses (e.g. Elemo, Satici, & Griffiths, 2020; Sakib et al., 2020; Winter et al., 2020). Others have found some evidence for multidimensionality though. For example, Bitan et al. (2020) and Reznik et al. (2020) used orthogonal rotation from factor analysis or principal components analysis for two dimensions and found that items I1 (most afraid), I2 (uncomfortable), I4 (losing my life) and I5 (nervous or anxious) loaded together and that items I3 (clammy), I6 (cannot sleep) and I7 (heart races) loaded together. These two factors (or components) differentiated emotional reactions to fear (the first four items) from physical symptoms of fear (the last three items). Pakpour et al. (2020), however, criticised these findings, arguing that the authors presented no theoretical rationale for testing a two-factor structure and that they should have used confirmatory factor analysis rather than exploratory factor analysis. The bigger concern from our perspective is that orthogonal rather than oblique rotation was used because there is no theoretical rationale for constraining the inter-factor correlation to zero.

Masuyama, Shinkawa and Kubo (2020) used confirmatory factor analysis to test a one-factor model and a two-factor model with the two factors representing those found by Bitan et al. (2020) and Reznik et al. (2020). The authors do not report on the inter-factor correlation coefficient. However, they do provide the Pearson’s correlation coefficient for the two scale scores and their respective reliability coefficients. The Pearson’s correlation coefficient was 0.45. Correcting this for unreliability produces a correlation coefficient of 0.59, leading to some questions about the viability of a two-factor model. Huarcaya-Victoria et al. (2020), for example, found an inter-factor correlation coefficient of 0.72 in their exploratory factor analysis, and Caycho-Rodríguez et al. (2020) found an inter-factor correlation of 0.89 in their confirmatory factor analysis. Masuyama et al. (2020) then used a bifactor model but unfortunately do not provide the relevant statistical indices (e.g. Rodriguez, Reise, & Haviland, 2016) to determine if there is sufficient evidence for group factors. Huarcaya-Victoria et al. (2020) applied the correct statistical indices to their bifactor model and found that there was little evidence for group factors.

Iversen et al. (2021) produced a more complete analysis of the FCV-19S. They firstly fit a one-factor model to the FCV-19S item responses using confirmatory factor analysis. The model fit was somewhat unsatisfactory, but it did improve after allowing item residuals to correlate. These residual correlation coefficients ranged from 0.22 to 0.51 (across five residual correlations). The largest residual correlations were between items I6 (cannot sleep) and I7 (heart races) and then between items I1 (most afraid) and I2 (uncomfortable). They then modelled a two-factor model consisting of items I1, I2 and I4 (losing my life) on factor 1 and items I3 (clammy), I5 (nervous or anxious), I6 and I7 on factor 2. These two factors were labelled cognitive fear (factor 1) and somatic fear (factor 2). Not surprisingly, the two-factor model showed better model fit. However, the inter-factor correlation coefficient was 0.84, suggesting that the two factors show little to no discriminant validity.

### Item fit

Several studies used the Rasch measurement model to investigate the FCV-19S item functioning. Sakib et al. (2020) found that all the items fit the Rasch model. Item I7 (heart races) did show some misfit although it did not appear to be degrading to the overall quality of measurement. Pang et al. (2020), in contrast, found that item I3 (clammy) showed misfit to the Rasch model. Winter et al. (2020) found some misfit for item I4 (losing my life). Satici, Gocet-Tekin, Deniz and Satici (2020), Elemo et al. (2020) and Caycho-Rodríguez et al. (2020) used the graded response model to investigate the item functioning of the FCV-19S. Unfortunately, these three studies do not report any item fit statistics. Caycho-Rodríguez et al. (2020) also analysed the two factors separately arguing that the model fit statistics for the two-factor model in their confirmatory factor analysis was better than the fit statistics for the one-factor model. This is flawed logic though because (1) the inter-factor correlation coefficient was 0.89, meaning that the two vectors are only separated by ≈ 27°, and (2) the authors did not take sampling error into account in their fit statistics (e.g. Cheung & Rensvold, 2001), and (3) over-reliance on cut-offs for fit statistics is dubious (Nye & Drasgow, 2011). Their graded response model results are therefore difficult to interpret. With respect to category functioning and fit, Winter et al. (2020) found a monotonic increase in average person measures across categories and no threshold disordering. Category response fit is not reported in their study though.

### Item locations

Ahorsu et al. (2020) found that item I1 (most afraid) was the most difficult item to endorse in their sample. In contrast to this, Sakib et al. (2020), Winter et al. (2020) and Pang et al. (2020) found that item I1 was the easiest item to endorse. These authors also found that items I3 (clammy), I6 (cannot sleep) and I7 (heart races) were generally the most difficult items to endorse. With respect to the graded response model, Satici et al. (2020) and Elemo et al. (2020) found that items I2 (uncomfortable) and I5 (nervous or anxious) were the easiest to endorse and that items I3 and I6 were the most difficult to endorse. Elemo et al. (2020) also found that item I1 was the easiest to endorse, although the beta coefficient for the first threshold of this item appears to be an unstable estimate, meaning that the mean item difficulty for item I1 should not be overinterpreted. It is difficult to explain the reason for these somewhat contradictory findings as it depends on various factors, such as cross-country differences in the construct, differences in samples used and IRT model and estimator used in the analyses, amongst others.

### Reliability

Reliability coefficients for the FCV-19S-scale scores are mostly satisfactory when using the instrument for routine screening and research. Ahorsu et al. (2020), for example, found a Cronbach’s alpha of 0.82. Satici et al. (2020) found a Cronbach’s alpha of 0.85 and a McDonald’s coefficient omega total of 0.85. Winter et al. (2020) found Cronbach’s alphas of 0.88 and 0.89 across two sample groups, whereas Perz et al. (2020) found a Cronbach’s alpha coefficient of 0.91. Huarcaya-Victoria et al. (2020) found a coefficient omega hierarchical of 0.81 for the general factor and coefficient omega hierarchical subscale of 0.15 and 0.27 for the two group factors.

### Summary

The FCV-19S has emerged as a popular instrument for the measurement of fear of coronavirus-19. However, there are some limitations identified in existing studies. For example, some researchers have found evidence for multidimensionality, whereas others have found support for a unidimensional structure. Researchers have also tended to sometimes not report sufficient statistical information to allow for a more complete understanding of the psychometric properties of the FCV-19S. Against this background, we set out to investigate the internal validity of the FCV-19S using the Rasch PCM to better understand the psychometric properties of the FCV-19S and also to shed some insight into the use of the FCV-19S in South Africa.

## Method

We used a quantitative cross-sectional survey research design in this study.

### Research participants

The sample comprised 159 participants obtained using non-probability convenience sampling. The mean age of the participants was 36.78 (median = 32, standard deviation [SD] = 11.79) and consisted of approximately an equal number of self-identified men (*n* = 70, 44.30%) and self-identified women (*n* = 86, 54.43%).^3^ Two participants identified as non-binary (1.27%). Most of the participants identified as white (*n* = 135, 85.99%) followed by black African (*n* = 13, 8.28%), Indian and/or Asian (*n* = 5, 3.18%) and mixed race (*n* = 4, 2.55%). The participants were asked to indicate all the home languages that they spoke. Most of the participants indicated that they spoke English at home (*n* = 109, 55.53%), followed by Afrikaans (*n* = 70, 35.53%), and a local South African language (*n* = 12, 6.09%). Six (3.05%) of the participants indicated that they also spoke an international language at home. The participants were generally employed when completing the questionnaire with 114 (71.70%) indicating that they had full-time employment and with 6 (3.77%) indicating that they were self-employed. Fifteen (9.55%) of the participants had part-time employment and 22 (14.01%) of the participants were either unemployed or retired. Unfortunately, we did not obtain data on which industries these participants were employed in and what level they were in their organisations. With respect to relationship status, 117 (73.58%) participants were either in a relationship or married and 42 (26.42%) were either single, divorced or windowed. Most of the participants had a university degree (*n* = 106, 67.52%) or a certificate or diploma (*n* = 23, 14.65%). Twenty-eight (17.83%) of the participants had a Grade 10 or a Grade 12. Lastly, we asked the participants to indicate if they felt that the coronavirus-19 and the subsequent lockdown had impacted on their life. Twenty-nine (19.46%) of the participants indicated that they *strongly agree*, followed by 47 (31.54%) who indicated that they *agree*, 58 (38.93%) indicated that they *somewhat disagree* and *somewhat agree*, 8 (5.37%) indicated that they *disagree* and 7 (4.70%) indicated that they *strongly disagree*.

### Research procedure

Participants were invited to complete the FCV-19S, which was hosted on Google forms. The questionnaire pack included a participant information sheet, informed consent form and the FCV-19S. We used social media platforms (e.g. Facebook, LinkedIn and WhatsApp) to invite adults over the age of 18 years to participate in the research. The participant information sheet and informed consent form explained the nature of the research, that participation was voluntary, and that participation was confidential and anonymous. A list of helpline contact details was provided on the participant information sheet. These included contact details for the South African Depression and Anxiety Group, Lifeline, and the Coronavirus South African Resource Portal. We also provided a website link to the Coronavirus South African Resource Portal and the World Health Organization for those participants who wanted more information on coronavirus-19. To facilitate transparency in the results, we placed our data online for other researchers to access. We informed participants that their depersonalised item responses (i.e. no biographical information) would be placed online and be open-access. Responses were only included if participants consented to having their depersonalised item responses placed online.

### Instrument

We used the FCV-19S. Details about its response format and scoring were reported previously in the article.

### Analysis

We used the Rasch PCM to investigate the internal validity of the FCV-19S. The PCM is a polytomous extension of the dichotomous Rasch model where item thresholds are allowed to vary between items (i.e. item thresholds are not constrained to be equal across items as is done in the Rating Scale Model). No slope parameters are estimated in the PCM. An attractive feature of the Rasch model is that threshold disordering can be investigated. This provides useful information on the usefulness of the response options for each item (Bond, Yan, & Heene, 2021). The unidimensional PCM assumes that the item responses for each scale are unidimensional and that there is no local dependence. It is necessary to show that these assumptions hold before applying the Rasch model as violations of these assumptions can lead to misleading parameter estimates (Bond et al., 2021; Christensen, Makransky, & Horton, 2017).

### Data preparation

We collapsed the five-point Likert-type scale to a four-point scale because too few respondents selected the *strongly agree* category (*n* = 25 across all seven items). Leaving in the *strongly agree* category would lead to spare data and incorrect parameter estimates (see Cheng et al., 2014). The *agree* and *strongly agree* categories were therefore collapsed. We also removed one participant who had an unusual response pattern using person fit statistics from the Rasch model. This led to our sample size of 159 participants.

### Item parameters and item fit

Winsteps version 4.7.0.0 (Linacre, 2019) uses joint-maximum likelihood estimation with a proportional curve fitting algorithm to obtain item and person parameter estimates (Linacre, 2004; Meyer & Hailey, 2012). The proportional curve fitting procedure allows for more robust parameter estimates when some response frequencies are rarely observed by participants (Linacre, 2004). We changed the default convergence criteria in Winsteps to be based on both the logit change size and residual change size in the iterations using Δ 0.001 as the convergence criteria. Use of stringent change criteria can lead to better precision of parameter estimates (Linacre, 2019). Item fit was investigated using infit and outfit mean squares. Mean square values > 1.30 (underfit) or < 0.70 (overfit) are usually used as criteria to determine potential item misfit (e.g. Bond et al., 2021). To supplement these item-level fit statistics, we used bootstrapping with 5000 resamples to obtain the sampling distribution of the infit and outfit statistic for each item (see Seol, 2016, for a discussion^4^). We then used 95% percentile confidence intervals to determine if the item fit statistics deviated from the expected value of 1.00 for each item.

### Dimensionality and local independence

We investigated unidimensionality of the item responses using Revelle’s unidimensionality test, a comparison of the first to second eigenvalue in the item correlation matrix, exploratory dimensionality evaluation of to enumerate contributing traits (DETECT) and bifactor confirmatory factor analysis. Revelle’s unidimensionality test computes the ratio of the observed item correlation matrix to the model-implied item correlation matrix from a one-factor model. The raw index replaces the observed correlation matrix with the communality coefficients from the factor model, whereas the adjusted index leaves the diagonal of the observed correlation matrix at unity for each item (Revelle, 2020).

The logic of the ratio of the first to second eigenvalue in a square correlation matrix (i.e. the observed correlation matrix) is that the first component should explain most of the variance if the item responses are unidimensional. This approach to investigation unidimensionality is not recommended (Roznowski, Tucker, & Humphreys, 1991), and therefore, we include it here as an exploratory statistic that can be used to supplement the results from the other statistical techniques. Principal components analysis of standardised residuals applies principal components analysis to the correlation matrix after removing the Rasch dimension. There should be no structure in the standardised residual correlation matrix if unidimensionality holds. Component loadings are contrasted on components that have structure (i.e. large eigenvalues) to determine if there is a meaningful secondary dimension in the data (Linacre, 2019; Smith & Miao, 1994).

Exploratory DETECT uses a conditional covariance matrix to find item partitions that maximise the DETECT index (Monahan, Stump, Finch, & Hambleton, 2007; Zhang & Stout, 1999). We used an exploratory rather than a confirmatory approach because the sample size is small, there are few items in the scale (see Zhang, 2007), and because we were interested in the clustering solution to determine which clusters were found in the conditional covariance matrix. Structure in the clustering solution would indicate the presence of a potential secondary dimension. We ran the DETECT statistic 5000 times using a different seed each time and then averaged the cluster loadings on the first cluster partition to investigate if items consistently loaded into different clusters. We then used this clustering solution to form the group factors in the confirmatory bifactor analysis. In general, the bifactor approach to testing unidimensionality should not be used when there is not a clear secondary dimension (Reise, Morizot, & Hays, 2007). However, we used this approach because other studies have found potential secondary dimensions and because the bifactor model can give a clear indication of variance decomposition between the general and group factors (Rodriguez et al., 2016). We used the explained common variance (ECV) and item ECV (I-ECV) to investigate the feasibility of forming a subscale score. An ECV > approximately 0.70 can be used as a tentative cut-off of unidimensionality (Quinn, 2014). We also calculated coefficient omega hierarchical and omega hierarchical subscale to determine the reliability of the general factor and the group factors (Rodriguez et al., 2016).

Local dependence was investigated using Yen’s Q3 statistic. The expected value of Yen’s Q3 for seven items is -0.17. We subtracted the average residual correlation from the Q3 correlation matrix and used a corrected correlation > |0.30| as a tentative indication of local dependence (Christensen et al., 2017). We exported the raw residuals from Winsteps and then calculated the correlation coefficients manually.

### Test and item information

Test and item information is similar to reliability in classical test theory and indicates the statistical information given the test and the items (DeMars 2010; Linacre, 2019). The standard error of measurement can be obtained by using the inverse square root of the information function (Linacre, 2019). The information function and standard error of measurement are used to determine the precision of estimated person scores across the logit range of the underlying trait for the test or items (DeMars 2010). This can be used to determine the measurement range of the test or items (Linacre, 2019).

### Software

We used Winsteps version 4.7.0.0 (Linacre, 2019) for the PCM calibration. All other analyses were conducted in *R* version 4.0.3 (R Core Team, 2020). Revelle’s unidimensional test was performed using the *psych* package version 2.0.7 (Revelle, 2020) and the DETECT statistic with the *sirt* package version 3.9-4 (Robitzsch, 2020). The *lavaan* package 0.6-7 (Rosseel, 2012) was used for the confirmatory bifactor analysis and the *BifactorIndiciesCalculator* package version 0.2.1 (Dueber, 2020) was used to calculate the bifactor indices. All figures were made by exporting the relevant data from Winsteps into *R* and using the default graphics package.

### Ethical considerations

Ethical clearance for the study was obtained from the Department of Industrial Psychology and People Management research ethics committee at the University of Johannesburg, reference number: IPPM-2020-451.

## Results

Item descriptive statistics and response frequencies for each item category (labelled C1–C4) are provided in Table 1. Inspection of this table shows that items I3, I6 and I7 were somewhat difficult to endorse in this sample because they had lower mean values and because their C4 category response options were seldom endorsed (even after the *agree* and *strongly agree* categories were collapsed).

**TABLE 1:** Item descriptive statistics and response categories frequencies.

Item	Mean	Median	SD	MAD	SEM	C1	C2	C3	C4
I1	2.51	2	1.04	1.48	0.08	0.18	0.37	0.22	0.23
I2	2.28	2	1.04	1.48	0.08	0.25	0.42	0.15	0.19
I3	1.65	2	0.69	1.48	0.06	0.46	0.45	0.07	0.02
I4	2.28	2	1.00	1.48	0.08	0.23	0.42	0.18	0.16
I5	2.50	2	1.11	1.48	0.09	0.22	0.33	0.18	0.27
I6	1.57	1	0.67	0.00	0.05	0.51	0.43	0.04	0.02
I7	1.72	2	0.84	1.48	0.07	0.47	0.40	0.08	0.06

MAD, median absolute deviation; SEM, standard error of the mean; C1-C4, response categories 1-4. Multiply the values in the response categories by 100 to get percentage responses in each category.

### Dimensionality

The raw ratio index and adjusted ratio index for Revelle’s unidimensionality test were 1.00 [1.00, 1.00] and 0.87 [0.83, 0.90].^5^ Eigenvalue decomposition of the item Pearson and polychoric correlation matrices produced a ratio of the first (Pearson *λ* = 3.91, polychoric *λ* = 4.62) to second (Pearson *λ* = 1.05, polychoric *λ* = 0.90) eigenvalues of 3.72 and 5.13. Principal components analysis of the standardised residuals from the PCM showed that there was one potential secondary dimension with an eigenvalue of 2.07. The mean eigenvalue of 5000 simulated data sets (i.e. simulated to fit the Rasch model) was 1.47 [1.34, 1.64], suggesting that the secondary dimension was larger than what would be expected given the model parameters. Inspection of the component loadings on this dimension showed that there was a contrast between items I3, I6 and I7 (contrast one) and items I1 and I4 (contrast two). The disattenuated correlation coefficient between these two contrasts was 0.74 (excluding extreme responses) and 0.94 (including extreme responses). This implies that the two clusters share approximately 55% to 88% variance. Exploratory DETECT showed that items I2, I3, I6 and I7 (cluster one) and items I1 and I4 (cluster two) generally clustered together. Item I5 did not consistently fall into either of these clusters.

For the confirmatory bifactor analysis, we allowed items I1 and I4 to load on the general factor and a group factor. The factor loadings of the two items were constrained to equality to allow for estimation. Items I2, I3, I6 and I7 were allowed to load on the general factor and a group factor. We allowed item I5 to only load on the general factor. The bifactor model showed satisfactory overall fit [χ^2^(9) = 20.175, *p* = 0.017, CFI = 0.97, TLI = 0.94, RMSEA = 0.09 (90% CI = 0.04, 0.14), USRMR = 0.03, MLM estimation was used in this analysis].^6^ The ECV of the general factor was 0.79 and the ECV of the group factors were 0.20 (items I1 and I4) and 0.34 (items I2, I3, I6 and I7). Six of the items had I-ECV values > 0.50. Item I6 had the lowest item ECV of 0.30, suggesting that the item I6 might be measuring something somewhat different to the other items. Notably, this item also had a much larger standardised factor loading on the group factor (*λ* = 0.53). Coefficient omega hierarchical was 0.77 and coefficient omega hierarchical subset was 0.16 (items I1 and I4) and 0.21 (items I2, I3, I6 and I7). The items in the two clusters above appeared to differentiate between psychological symptoms of fear (items I1 and I4) and physical symptoms of fear or anxiety (items I2, I3, I6 and I7).^7^ Iversen et al. (2021) used the terms cognitive fear and somatic fear for their two factors.

The mean Yen’s Q3 statistic was -0.16. Subtracting the mean correlation from the Q3 correlation matrix showed that three item pairs had potential local dependence when using |0.30| as a cut-off (*r* item I1 and I4 = 0.30, *r* item I3 and I6 = 0.39, *r* item I6 and I7 = 0.54). Local dependence was particularly problematic for items I6 and I7 as it translates to approximately 29% shared variance in the conditional correlation coefficients.

### Model and item fit

The Rasch PCM model produced a χ^2^ of 1669.19 on approximately 1668 degrees of freedom with a *p* value of 0.487. The model RMSR^8^ was 0.588 and the expected RMSR was 0.591. The standardised residuals had a mean of -0.01 and an SD of 1.00. These statistics suggest satisfactory overall model fit. The satisfactory model fit was supported by the mean person and item outfit and infit mean squares of 0.99 and 1.04 (person) and 0.99 and 0.99 (item). Item fit statistics are provided in Table 2.

**TABLE 2:** Item locations and fit statistics.

Item	Location	Outfit MNSQ		*Z*	Infit MNSQ		*Z*
		Mean	95% CI		Mean	95% CI	
I1	−1.32	1.15	0.86, 1.50	1.13	1.05	0.84, 1.30	0.51
I2	−0.72	1.16	0.86, 1.50	1.17	1.15	0.89, 1.40	1.25
I3	1.46	1.02	0.82, 1.26	0.20	1.10	0.91, 1.31	0.81
I4	−0.70	1.26	0.94, 1.59	1.90	1.09	0.87, 1.34	0.82
I5	−1.16	0.98	0.73, 1.29	−0.08	0.88	0.69, 1.09	−1.16
I6	1.63	0.83	0.68, 0.99†	−1.04	0.96	0.78, 1.13†	−0.23
I7	0.80	0.58	0.48, 0.69†	−3.00	0.69	0.57, 0.82†	−2.55

MNSQ, mean square.

† , mean squares do not include 1.00 in the 95% confidence intervals.

The infit mean squares ranged from 0.69 (item I7) to 1.15 (item I2) and the outfit mean squares ranged from 0.58 (item I7) to 1.26 (item I4). The items generally showed satisfactory model fit with all of the 95% percentile bootstrapped confidence intervals, except for item I7, intersecting with 1.00. Item I7 was also the only item to show statistically significant misfit when using the *Z* value. This misfit remained statistically significant after applying a Bonferroni correction (outfit *p* = 0.020, infit *p* = 0.035). Item characteristic curves for the seven items are presented in the Online Supplement. Inspection of these figures supports the relatively large overfit for item I7. Outfit and infit mean squares for the item response categories are presented in Table 3. Inspection of this table shows that the third response categories generally showed misfit (Items I1, I3 and I4), although these fit statistics should not be overinterpreted given the relatively sparse data. Items I2, I5 and I7 showed disordered Rasch–Andrich rating scales. However, the expected person measures did increase monotonically across all the response categories, meaning that the Rasch model assumption of monotonically increasing person parameters was not violated.

**TABLE 3:** Item response categories fit and thresholds.

Response categories	Observed Average	Sample Expected	Observed – Expected	Outfit	Infit	Rasch–Andrich	Thurstone
**Item 1**							
C1	−3.05	−3.11	0.06	1.02	0.94	-	-
C2	−1.29	−1.44	0.15	1.11	1.09	−2.56	−3.92
C3	−0.61	−0.26	−0.35	1.48	1.48	1.06	−0.61
C4	0.80	0.72	0.08	1.07	0.91	1.51	0.55
**Item 2**							
C1	−2.67	−2.72	0.05	1.10	1.26	-	-
C2	−1.00	−1.04	0.04	1.08	1.10	−2.18	−2.93
C3	0.00	−0.04	0.04	0.91	0.89	1.24	−0.01
C4	0.76	0.91	−0.15	1.40	1.21	0.94	0.80
Item 3							
C1	−1.77	−1.85	0.08	1.20	1.24	-	-
C2	−0.25	−0.21	−0.04	0.65	0.89	−2.66	−1.21
C3	0.83	1.00	−0.17	1.34	1.17	0.79	2.06
C4	2.36	2.22	0.14	0.86	0.92	1.86	3.57
**Item 4**							
C1	−3.07	−2.77	−0.30	0.79	0.67	-	-
C2	−0.79	−1.06	0.27	1.26	1.18	−2.36	−3.09
C3	−0.38	−0.01	−0.37	2.31	1.75	1.04	−0.03
C4	0.92	0.98	−0.06	0.96	0.97	1.31	1.02
**Item 5**							
C1	−3.04	−2.91	−0.13	0.83	0.64	-	-
C2	−1.25	−1.31	0.06	1.02	0.88	−1.97	−3.18
C3	−0.45	−0.28	−0.17	1.10	1.17	1.06	−0.58
C4	0.74	0.65	0.09	0.97	0.89	0.92	−0.28
Item 6							
C1	−1.79	−1.72	−0.07	1.00	0.98	-	-
C2	0.00	0.10	0.10	0.59	0.93	−2.51	−0.89
C3	0.81	1.13	−0.32	1.15	1.16	1.14	2.39
C4	2.74	2.31	0.43	0.46	0.68	1.37	3.43
Item 7							
C1	−2.01	−1.84	−0.17	0.87	0.86	-	-
C2	−0.26	−0.30	0.04	0.34	0.50	−1.85	−1.10
C3	1.20	0.68	0.52	0.39	0.48	1.05	1.36
C4	1.94	1.73	0.21	0.66	0.78	0.80	2.17

Note: Observed Average, average person logits (excluding extreme responses) for each category; Sample Expected, expected person logits for each category; Observed–Expected, observed average logits - expected logits; Rasch–Andrich, Rasch–Andrich thresholds; Thurstone, Rasch–Thurstone thresholds. Category labels (C1–C4) refer to each response category.

### Item difficulty

**FIGURE 1:**
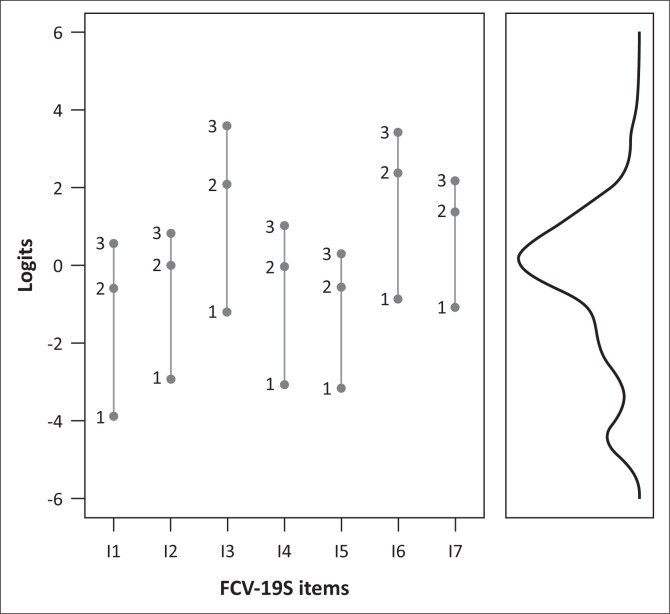
Wright map with Rasch–Thurstone thresholds reported on the left and the person measure density plot on the right.

In Table 2 the mean item locations are given, and in Table 3 the Rasch-Andrich thresholds and Thurstone thresholds are given. The Rasch–Andrich thresholds are reported as threshold + mean item location, whereas the Thurstone thresholds are not.^9^ Item I1 was the easiest item to endorse (mean location = -1.32) and item I6 was the most difficult item to endorse (mean location = 1.63). A Wright map for the *Thurstone* thresholds is presented in Figure 1. Inspection of this figure shows that the items covered a fairly wide range of the latent trait although there were about 18 participants with person measures below the lowest Thurstone threshold (item I1). Category locations for each item are presented in Table 3.

### Test and item information and reliability

**FIGURE 2:**
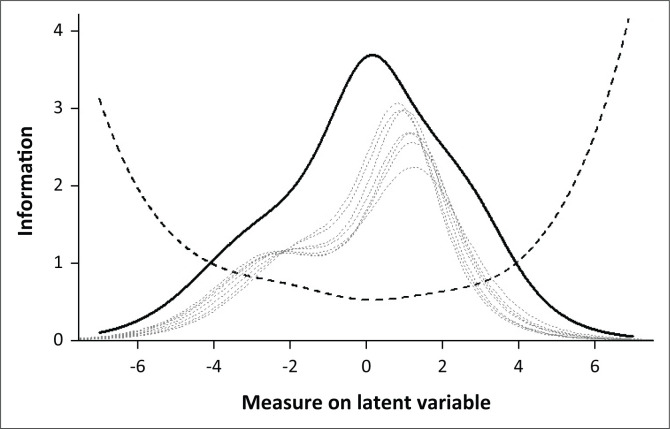
Test information function and standard error of measurement. Item information functions given as dashed grey lines. These information indices were scaled by 4 on the *Y*-axis for ease of plotting.

The scale provided the most information between approximately -4.00 and 4.00 logits and peaked at approximately 0.16 logits. The maximum item information ranged from 0.53 (item I3) to 0.76 (item I7). The test and item information functions are provided in Figure 2. The marginal reliability of the items was 0.87. This compared well to the Cronbach’s alpha coefficient of 0.86 [0.82, 0.89] and the coefficient omega total coefficient of 0.86 [0.80, 0.89].

### Relationship with biographical variables

In the Online Supplement, we provide correlation coefficients for the summated FCV-19S scale scores and relevant biographical characteristics previously reported for the reader who is interested.

## Discussion

This study set out to investigate the internal validity of the FCV-19S in South Africa using the Rasch measurement model. Our results showed that the FCV-19S item scores are essentially unidimensional. There is some evidence for the psychological and physical aspects of fear somewhat loading on separate dimensions although the strength of their respective group factors appears to be too small to warrant forming two separate scales. These results support those obtained by Huarcaya-Victoria et al. (2020) and show that the two dimensions found by Bitan et al. (2020) and Reznik et al. (2020) are not substantively important in the current FCV-19S. Items I6 (cannot sleep) and I7 (heart races) showed large local dependence. Iversen et al. (2021) also found that these two items had large residual correlations. This means that these two items are correlated after conditioning on the general factor and that one of the items in the item pair may be redundant (see Zenisky, Hambleton, & Sireci, 2001, for a discussion of these issues).

Most of the FCV-19S items in our analysis showed satisfactory fit to the Rasch model. Item I4 (losing my life) did show some minor underfit although it was not large enough to be of concern. Winter et al. (2020) also found some misfit for item I4. Item I7, in contrast, showed statistically significant and practically large overfit. Overfit is not necessarily as big a concern as underfit because it implies that the expected item responses are too predictable. This can have the unintended consequence of artificially increasing reliability estimates (Linacre, 2019). Sakib et al. (2020) found that item I7 showed underfit rather than overfit. However, given our small sample size, we would not overinterpret the differences between our results and their results. At the category level, we found some misfit for the third response category for most of the items. However, our small sample size led to sparse data, and these results should not be overinterpreted (see Cheng et al., 2014). Three of the items, namely, I2 (uncomfortable), I5 (nervous or anxious) and I7, showed disordered Rasch–Andrich thresholds. This means that some of the response categories measure a narrow interval of the underlying trait and might not be needed (Linacre, 2019). Winter et al. (2020), in contrast, found no threshold disordering in their study.

We found that Item I1 (most afraid) was the easiest item to endorse and that items I3 (clammy) and I6 were the most difficult to endorse. Similar results were obtained by Satici et al. (2020), Elemo et al. (2020), Winter et al. (2020) and Pang et al. (2020).^10^ Based solely on item content, it makes substantive sense that this should be the easiest item to endorse. It is therefore unclear why Ahorsu et al. (2020) found that it was one of the most difficult items in their scale. The most difficult items to endorse in our sample group were items I3, I6 and I7. These results were again also found by Winter et al. (2020) and Pang et al. (2020). None of the items were too easy or too difficult to endorse. This can be seen in the Wright map and the test information function, and implies that the FCV-19S is a reliable measure of fear across a wide range of the underlying person location distribution. That said, the Wright map did show that some people had person measures that were outside of the lowest item Thurstone threshold. We discuss the implications of these results under the next heading.

The reliability of the FCV-19S in our study was somewhat satisfactory if the FCV-19S is used as a general screening tool (i.e. not to make diagnoses). Our reliability coefficients generally matched those obtained in other studies (e.g. Ahorsu et al., 2020; Huarcaya-Victoria et al., 2020; Perz et al., 2020). It must be kept in mind though that the local dependence between items I6 and I7 and the large overfit for item I7 might have artificially inflated reliability in this study.

### Implications for research and practice

Our results hold several implications for further development of the FCV-19S. Firstly, the authors might want to consider adding more items to the scale. This could be performed if a distinction wants to be made between the psychological and physical components of fear and to increase the reliability of the scale. In addition, practical implications should be considered (i.e. what is the intended use of the scale). Adding items to increase dimensionality would require careful consideration though because appropriate analyses (i.e. bifactor modelling) would need to be conducted to determine if it is warranted to create two separate subscale scores. Attention should also be given to the local dependence between items I6 (cannot sleep) and I7 (heart races). It is not clear if this is a sample-specific result or if it will generalise across different studies. Replication of this dependency would suggest that either of these two items are redundant and should be reworded or removed. Item I7 seems to be the best candidate in this regard because the item’s wording consists of metaphorical language (*races*) and jargon (*palpitates*). The two items also have mostly overlapping thresholds.

At the category level, researchers should consider the value of using a five-point Likert-type scale. There might be value in using fewer response categories (such as the four-point scale) to make responding to the items easier, especially given the disordered Rasch–Andrich thresholds. However, this reduction comes at the cost of reduced item information (Linacre, 2019). Researchers might want to also consider adding some easier items to the FCV-19S to capture those who score particularly low on fear (see Figure 1). This can form part of the general strategy of writing new items for the scale. However, this will depend on the use of the scale scores and will only be necessary if researchers are interested in those who score particularly low, which is likely not the intended audience of the scale. A potential solution to the aforementioned problems is to create a larger pool of items that researchers could then choose from depending on the purpose of their research.

For practice, our results generally support the use of the FCV-19S scores in this sample group. Given the small sample size and convenience sample, it would be difficult to make a general conclusion of the suitability of the FCV-19S for the South African context. For example, our sample consisted of mostly English-speaking participants who generally identified as white and reported having quite a high level of education on average. We therefore cannot, based on one small sample group and one study, make a recommendation on whether the FCV-19S should be used in practice. This decision should be made by the practitioner taking all evidence into account.

Although we have not commented on this until now, researchers and practitioners in South Africa should consider the wording of the FCV-19S items. For example, item I5 (nervous or anxious) uses the term *social media*. This assumes that everyone completing the FCV-19S has access to social media. It might help to change this item to include *social media* and/or on television (e.g. news reports or newspapers). Item I7 (heart races) also uses the term *palpitates*, which is a complicated term (or as previously indicated, jargon). There might be value in reducing the complexity of the language used in the FCV-19S items to make the items more applicable to people regardless of their language or reading ability. Lastly, the FCV-19S uses the term coronavirus-19. It might be easier for participants if the term covid is used instead, as this is how people generally refer to the coronavirus-19, although this possibility would have to be more fully investigated.

### Limitations

We have already mentioned the limitation of the sample group we used in our study. This limitation has implications for the generalisability of the results in South Africa. A much larger sample size that is more representative of the general South African population is required to make a definitive conclusion about the psychometric properties of the FCV-19S in South Africa. That said, our results do serve as an initial source of evidence that could be built on in future research. Small sample sizes also require careful consideration because parameter estimates can become less stable. This can be seen in the relatively large standard errors for some of the parameter estimates making point estimates less certain. A proper measurement invariance study would also be required to determine if the items function the same across different groups (e.g. those who have been diagnosed as having coronavirus-19 and those who have not, participants from different language groups, etc.). We call for a proper replication of our results to better understand the psychometric properties of the FCV-19S in South Africa.

## Conclusion

This study set out to investigate the fit of the FCV-19S items to the Rasch measurement model in South Africa and to address some of the limitations and mixed findings identified in the previous research. Our results show support for the use of the FCV-19S in South Africa although much more research is needed before any definitive conclusions can be made about its psychometric properties.

## Footnotes

1 This is not a complete list of all studies investigating the psychometric properties of the FCV-19S.

2 We also only report on the internal validity results from these studies. Many of these studies investigated external validity, but this was not of interest in our study.

3 All percentages in this section are based on the number of complete cases and therefore do not include missing values.

4 We used bootstrapping of the participant responses rather than simulation of data based on the model parameters in this study.

5 All confidence intervals unless otherwise noted are 95% bootstrapped confidence intervals.

6 CFI, comparative fit index; TLI, Tucker–Lewis index; RMSEA, root mean square error of approximation; USRMR, unbiased standardised root mean residual (Maydeu-Olivares, Shi, & Rosseel, 2018); MLM, robust maximum likelihood estimation. Ninety per cent normal theory confidence intervals for the RMSEA reported in parentheses.

7 We also modelled a correlated two-factor solution excluding item I5 and found an inter-factor correlation coefficient of 0.67. We then modelled another bifactor model with item I2 moved to the psychological symptoms factor (as done by Iversen et al., 2021). However, item I2 had a negative factor loading on the group factor in this model.

8 Root mean square residual.

9 Subtract the mean item location from the Rasch–Andrich threshold to facilitate comparison to the Thurstone thresholds.

10 Item locations (i.e. the actual values) should not be directly compared between studies although the relative ordering can be compared because item locations should remain invariant within a linear transformation (DeMars, 2010).
